# Measles and rubella seroprevalence in adults using residual blood samples from health facilities and household serosurveys in Palghar District, Maharashtra, India, 2018 – 2019

**DOI:** 10.1017/S0950268824001389

**Published:** 2024-12-06

**Authors:** Christine Prosperi, Alvira Z. Hasan, Amy K. Winter, Itta Krishna Chaaithanya, Neha R. Salvi, Sanjay L. Chauhan, Ragini N. Kulkarni, Abhishek Lachyan, Poonam Gawali, Mitali Kapoor, Vaishali Bhatt, Ojas Kaduskar, Gururaj Rao Deshpande, Ignacio Esteban, Sabarinathan Ramasamy, Velusamy Saravana Kumar, Shaun A. Truelove, Muthusamy Santhosh Kumar, Jeromie W. Vivian Thangaraj, Lucky Sangal, Sanjay M. Mehendale, Gajanan N. Sapkal, Nivedita Gupta, Kyla Hayford, William J. Moss, Manoj V. Murhekar

**Affiliations:** 1 International Vaccine Access Center, Department of International Health, Johns Hopkins Bloomberg School of Public Health, Baltimore, MD, USA; 2 Department of Epidemiology, Johns Hopkins Bloomberg School of Public Health, Baltimore, MD, USA; 3 Department of Health Research, Model Rural Health Research Unit, Dahanu, India; 4 Indian Council of Medical Research, National Institute for Research in Reproductive and Child Health (NIRRCH), Mumbai, India; 5 Diagnostic Virology Group, Indian Council of Medical Research, National Institute of Virology, Pune, India; 6 Indian Council of Medical Research (ICMR), National Institute of Epidemiology, Chennai, India; 7 World Health Organization, Southeast Asia Region Office, New Delhi, India; 8 PD Hinduja Hospital and Medical Research Centre, Mumbai, India; 9 Division of Epidemiology and Communicable Diseases, Indian Council of Medical Research, New Delhi, India

**Keywords:** measles (rubeola), rubella, serology, vaccines

## Abstract

Residual blood specimens collected at health facilities may be a source of samples for serosurveys of adults, a population often neglected in community-based serosurveys. Anonymized residual blood specimens were collected from individuals 15 – 49 years of age attending two sub-district hospitals in Palghar District, Maharashtra, from November 2018 to March 2019. Specimens also were collected from women 15 – 49 years of age enrolled in a cross-sectional, community-based serosurvey representative at the district level that was conducted 2 – 7 months after the residual specimen collection. Specimens were tested for IgG antibodies to measles and rubella viruses. Measles and rubella seroprevalence estimates using facility-based specimens were 99% and 92%, respectively, with men having significantly lower rubella seropositivity than women. Age-specific measles and rubella seroprevalence estimates were similar between the two specimen sources. Although measles seropositivity was slightly higher among adults attending the facilities, both facility and community measles seroprevalence estimates were 95% or higher. The similarity in measles and rubella seroprevalence estimates between the community-based and facility serosurveys highlights the potential value of residual specimens to approximate community seroprevalence.

## Introduction

Serological surveys that test for measles and rubella virus-specific immunoglobulin G (IgG) antibodies can be used to estimate population seroprevalence. Assuming seropositivity is a correlate of protection, seroprevalence provides estimates of population immunity and its complement susceptibility. Immunity to measles and rubella is achieved from either past infection or successful vaccination. Immunity profiles typically vary over age, time, and space, depending on vaccine coverage and effectiveness, and factors that impact virus transmission such as birth rate, population size, and effective contacts over age and space. Knowledge of susceptibility profiles across demographic characteristics are critical for informing strategies to control the spread of measles and rubella viruses [1].

There are many challenges in conducting serological surveys (serosurveys), including the need for laboratories and laboratory expertise to accurately perform the assays. Population-based serological surveys, the gold standard, are expensive and time-consuming, requiring expertise to construct a sampling frame and to conduct the sampling across the population of interest [2, 3]. Given the necessary time commitment, population-based serosurveys are often cross-sectional, and by the time the sampling, testing, and analysis are completed, the immunity profile of the population may have changed. One strategy to increase the feasibility of generating and using these rich data is to rely on residual specimens from health facilities. However, the potential bias of serosurveys using residual specimens from health care facilities is unknown and likely to vary by setting [4].

There are situations where the use of residual specimens from health facilities is of value. The evaluation of rubella-virus-specific IgG antibodies among pregnant women during prenatal care visits is used to assess the potential for congenital rubella infection of the fetus. Rubella virus infection among children is generally a mild infection; however, rubella virus infection among pregnant women can lead to spontaneous abortion, fetal death, and the birth of a child with a suite of birth defects known as congenital rubella syndrome. Data from routine testing are often published, based on the underlying assumption that the seroprevalence estimates from routine prenatal testing is a good representation of all women of reproductive age [5]. This assumption, to our knowledge, has never been tested in populations of interest. Measles serological testing in health facilities is not routinely done other than among healthcare workers, nor are there any known studies assessing the use of health facility samples for measles serosurveys.

Residual health facility samples may be a promising source for conducting serosurveys among adults, a population often neglected in serosurveys despite the potential for immunity gaps in some epidemiological settings [6, 7]. We are aware of only two prior studies comparing seropositivity between adult community-based and residual specimen-based serosurveys, both conducted for severe acute respiratory syndrome coronavirus 2 (SARS-CoV-2) in urban US settings [8, 9]. In this study, we estimated seroprevalence to measles and rubella viruses using residual specimens collected from individuals 15 – 49 years of age attending sub-district hospitals in Palghar District, Maharashtra, India, during 2018 – 2019. The first and second doses of measles-containing vaccine (MCV) were introduced into the Universal Immunization Programme in India, in 1985 and 2010, respectively. At the time of our study, individuals ~33 years and younger would have had at least one opportunity to receive MCV. None of the individuals in this age group would have been eligible for the 2018 – 2020 measles-rubella (MR) vaccination campaign, which targeted children 9 months to 15 years of age and only those accessing vaccines in the private sector would have had access to a rubella-containing vaccine (RCV). We assessed the utility of residual specimens to identify immunity gaps in adults by comparing these results with data from a concurrent community-based serosurvey. We also explored whether seroprevalence estimates for subgroups, such as males or antenatal care (ANC) attendees, were representative of the population.

## Methods

Anonymized residual specimens from female and male adults aged 15 – 49 years seen at the two sub-district hospitals in Dahanu and Kasa in Palghar District, Maharashtra, India were collected from 27 November 2018 to 5 March 2019. The facility was requested to retain all blood specimens after testing. Study staff, based at the local Model Rural Health Research Unit (MRHRU), selected the first five specimens per day from the set of eligible adult specimens. Specimens were excluded if age was not available, if received by the study staff after more than 96 h of collection from the patient, or if there was no visible serum in the tube. Linked data, including age, sex, and whether the female patient was attending an ANC visit, were abstracted from the facility records.

Women aged 15 years to 49 years of age were enrolled in a district-representative, cross-sectional community-based serosurvey in Palghar District [10]. Thirty villages or wards in the district were selected based on the 2011 census using probability proportional to size systematic sampling method and one census enumeration block (CEB) was randomly selected from each. All individuals in the CEB were enumerated and 13 women per CEB were randomly selected. A venous blood sample and information on sociodemographic characteristics and history of vaccination with a RCV were collected after obtaining informed consent. The community-based serosurvey occurred 2 – 7 months after the residual specimen collection (26 April 2019 – 19 June 2019). No major changes in measles or rubella seroprevalence among women in this age group were expected during this time, as the supplemental immunization activity excluded individuals older than 15 years of age and there was no known measles or rubella outbreak.

The residual specimens were centrifuged at the sub-district hospital (3000 rpm for 10 min) and stored at 4 – 8°C in cold boxes until transported to the MRHRU laboratory where sera were aliquoted and stored at −20°C within 24 h of collection. The community-based blood specimens were also processed as sera at the MRHRU laboratory within 24 h of collection. All specimens were transported to the Indian Council of Medical Research (ICMR) National Institute of Virology in Pune in a cold box with dry ice. Sera were tested for IgG antibodies against measles and rubella viruses using the Euroimmun quantitative IgG enzyme immunoassay (Euroimmun AG, Lübeck, Germany; measles product Code: EI 2610-9601G; rubella: EI 2590-9601G) following the manufacturer’s instructions. Blood specimens from both surveys were tested in the same laboratory using the same kits; however, testing occurred at different times and with different kit lots. One of the measles kit calibrators changed between lots, which had an impact on lower quantitative results around the threshold for seropositivity. A linear correction derived from a lot-to-lot comparison was applied to the seropositivity estimates for the residual specimens to enable comparisons as previously described [10].

A sample size of ~450 residual specimens per group detects a difference of 5% between subgroups of interest (e.g., sex, ANC attendance) assuming a seroprevalence of 90% among adults with 80% power and 0.05 significance; however, the final sample size was dependent on the availability of specimens. Seroprevalence estimates for IgG antibodies against measles and rubella viruses were estimated with 95% confidence intervals. We explored differences in seroprevalence by sex with descriptive summaries and using logistic regression adjusted for age in years due to differences in the age distribution across the two serosurveys (Supplementary Figure S1). We also explored differences by whether the patient was seen for ANC. For this analysis, we included males and females in the non-ANC patient group, reflecting how residual specimen collection may be operationalized in a health facility (i.e., by not limiting non-ANC specimens to males or females only). The community-based serosurvey estimates were calculated using sampling weights based on survey design and accounting for non-response. We fit fractional polynomial models to the data to estimate measles and rubella age-specific seroprevalence.

To assess potential biases of the geographic distribution of the facility-based specimens, we evaluated measles and rubella seroprevalence spatial autocorrelation in the community samples collected in Palghar District, Maharashtra, and estimated a global Moran’s I statistic with over 1000 permutations using the R package spdep (version 1.2-5). We created distance-based spatial weight matrices based on a fixed distance value, based on the geographic extent of the spatial results and the minimal distance needed for all clusters to be included in the analysis (13 km). A Moran’s I statistic above 0 with a *p*-value < 0.05 indicates a spatial correlation between cluster seroprevalence. Analyses were performed using R (version 3.6.1).

The Institutional Ethics Committees of ICMR-National Institute of Epidemiology, Chennai, India, Johns Hopkins Bloomberg School of Public Health, Baltimore, USA, and ICMR-National Institute for Research in Reproductive and Child Health, Mumbai, India, approved the protocol. For the facility serosurvey, there was no interaction with human subjects and all specimens were deidentified. For the community-based serosurvey, written informed consent was obtained from all women aged 15 − 49 years before participation in the survey.

## Results

Six hundred and fifty specimens were collected from patients aged 15 through 49 years cared for at two sub-district hospitals in Palghar District, Maharashtra (Dahanu *N* = 344; Kasa *N* = 306). Seventy-three per cent were collected from female patients. Of the 476 female patients, 35% were seen for ANC services. The median age of all patients was 27 years (interquartile range: 22, 35). The male and non-ANC female patients were slightly older (median ages 30 and 29 years, respectively) than the ANC attendees (median age 24 years) (Supplementary Table S1).

**Figure 1.**
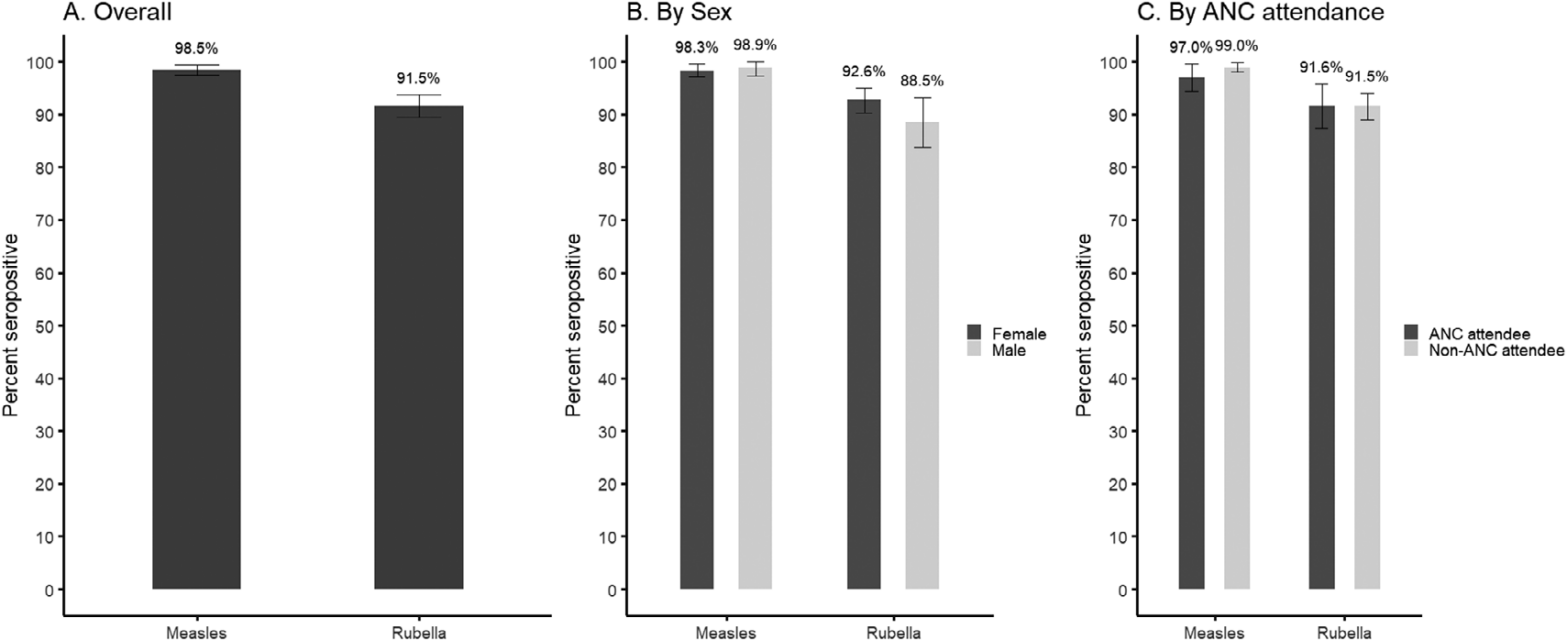
Measles and rubella seroprevalence among residual specimens collected from adult patients at health facilities. Significant difference for male vs. female for rubella seropositivity after adjusting for age in years (*p* = 0.04). Non-antenatal care (ANC) attendees includes male and female patients.

Ninety-nine per cent of patients had measles-virus-specific IgG antibodies (Figure 1 and Supplementary Table S2). There was no difference in measles seropositivity by sex or between ANC attendees and non-ANC patients. Rubella-virus-specific IgG antibodies were detected in 92% of patients (Figure 1 and Supplementary Table S2). Males had significantly lower rubella seropositivity compared to females (odds ratio [OR] adjusted for age: 0.54, 95% confidence interval [95% CI]: 0.30, 0.99). There was no difference in rubella seropositivity between ANC attendees and non-ANC patients.

**Figure 2.**
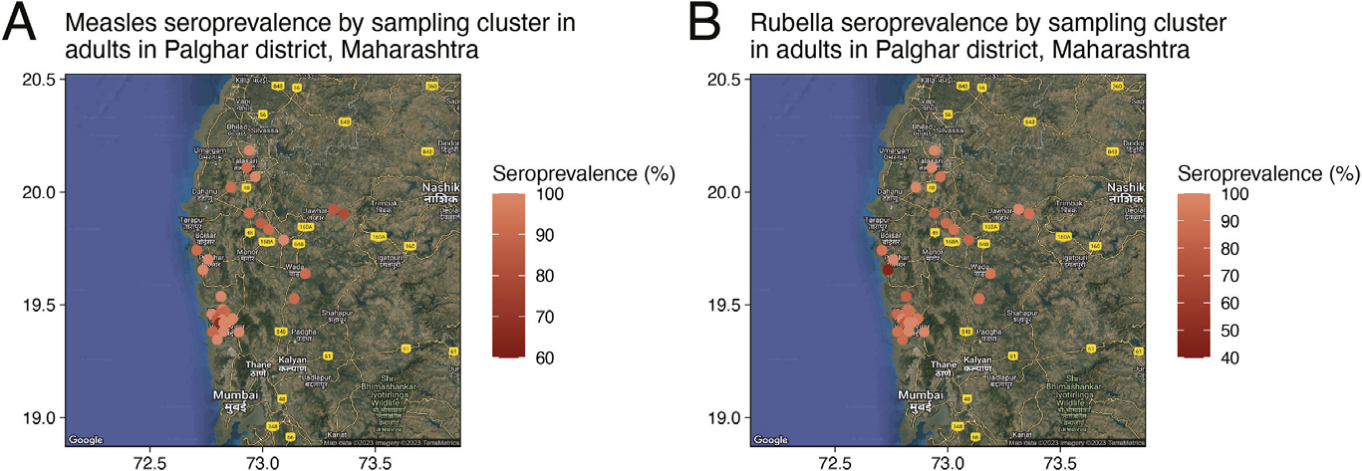
Spatial analysis of community survey measles and rubella seroprevalence in adults in Palghar District, Maharashtra. (a) Measles seroprevalence by sampling cluster. (b) Rubella seroprevalence by sampling cluster.

**Figure 3.**
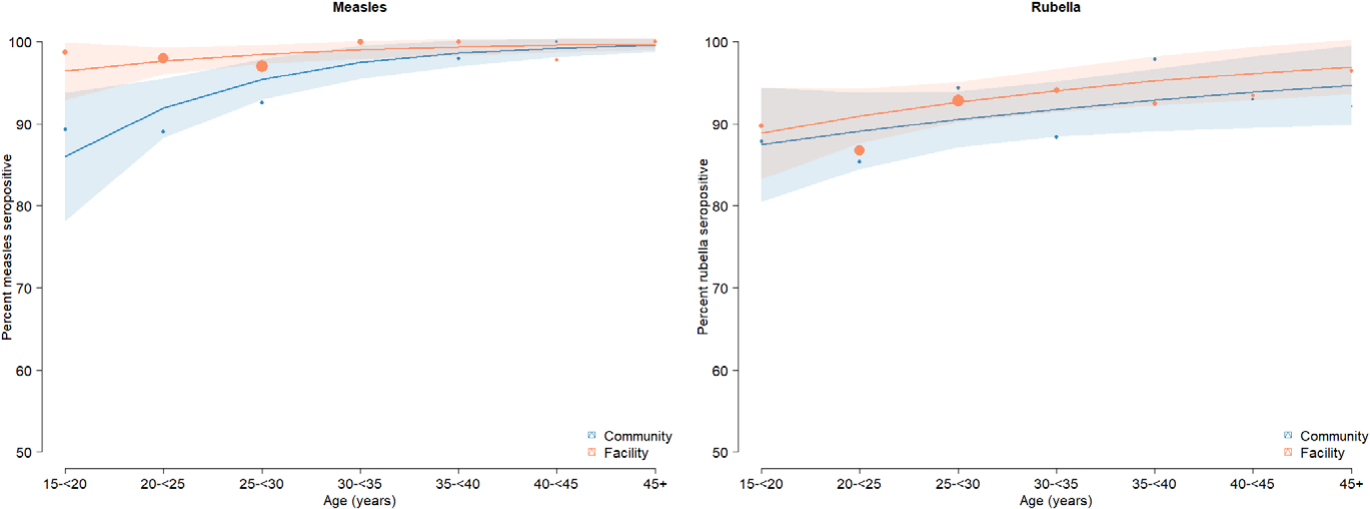
Measles and rubella age-specific seroprevalence estimated from residual and community adult specimens. Fitted line based on fractional polynomial models. Size of dots scaled by the number of specimens in each age bin. All adult residual specimens are included in these analyses (male and female).

In the community-based serosurvey, 94.9% of adult females were measles seropositive (95% CI: 92.1, 97.6) and 91% were rubella seropositive (95% CI: 87.2, 94.8) (Table 1). One-quarter of adult female participants reported a history of rubella vaccination (24.5%, 95% CI: 21.8, 27.2), treating unknown vaccination status in 38 women as not vaccinated. As there was no evidence of spatial heterogeneity in measles or rubella seropositivity within the district (Figure 2 and Supplementary Table S3), all residual and community serosurvey specimens were included in the comparison between survey designs. Adult females enrolled in the community serosurvey were slightly older than those attending the health facility (median age 30.8; Supplementary Figure S1). Age-specific measles and rubella seroprevalence for adults were similar between the two specimen sources for all age categories with the exception that measles seropositivity was higher among adults aged 15 − 30 years attending the facility (97.7%, 95% CI: 96.3, 99.2) compared to those enrolled in the community serosurvey (90.5, 95% CI: 85.3, 95.8%) (Figure 3). After adjusting for age, no difference in rubella seropositivity was observed when comparing residual specimens to those collected in the community serosurvey (Table 1). However, measles seropositivity was higher among patients with residual specimens after adjusting for age, although measles seroprevalence was 95% or higher using both specimen types. These findings comparing specimen types were consistent after restricting the residual specimens to females, ANC attendees, and male and non-ANC female patients (Table 1).

**Table 1.** Seroprevalence of immunoglobulin G antibodies against measles and rubella viruses among women aged 15 – 50 years enrolled in community-based survey and comparison with residual specimens

	Seroprevalence % (95% confidence interval)	Comparison with residual specimens *p*-value^a^			
Antigen	Community (*N* = 314)	Model 1: Community (*N* = 314) vs. all facility (*N* = 650)	Model 2: Community (*N* = 314) vs. all female facility (*N* = 476)	Model 3: Community (*N* = 314) vs. ANC attendees facility (*N* = 167)	Model 4: Community (*N* = 314) vs. male and non-antenatal care female patient facility (*N* = 483)
Measles	94.9 (92.1, 97.6)	0.01	0.01	0.07	**0.005**
Rubella	91.0 (87.2, 94.8)	0.32	0.34	0.70	0.32

*Note:* Community represents survey-weighted estimates for all clusters in the post-SIA survey.

a Logistic regression adjusted for age in years, with survey weights applied for community-based specimens. Models including both sex for residual specimens also adjusted for sex (Model 1 and Model 4). Bold indicates *p*-value < 0.006 (Bonferroni-adjusted *p*-value accounting for eight comparisons).

## Discussion

Using residual blood specimens collected from adults at two health facilities as well as specimens from a community-based serosurvey conducted nearly concurrently, we explored whether residual health facility specimens could identify immunity gaps in the overall adult population or specific subgroups (i.e., ANC vs. non-ANC samples). Measles and rubella seroprevalence from the two survey designs were similar and there were no differences across subgroups of residual samples, except for a slightly lower rubella seroprevalence among males.

Our primary aim was to evaluate the utility of residual specimens from health facilities to identify immunity gaps in a population of interest. We did not observe immunity gaps using either the community or facility specimens as measles and rubella seroprevalence from both sources was >90% among adults 15 − 49 years old. There was no statistically significant difference in rubella seroprevalence estimated from the two specimen sources. Although we did observe slightly higher measles seropositivity among adults attending the facility relative to the community, both facility and community measles seropositivity estimates were 95% or higher and the only statistically significant difference was the comparison between community specimens and residual specimens restricted to male and non-ANC female patients. However, when comparing measles seroprevalence from non-ANC to ANC subpopulations of residual specimens, there was no statistically significant difference. Thus, we conclude that measles seroprevalence estimates from the community and health facility serosurveys were similar. This similarity in measles and rubella seroprevalence between the two specimen sources supports the utility of residual specimens from health facilities to confirm high seroprevalence in this population, although we were not able to evaluate the potential to identify immunity gaps given the lack of such gaps in this community.

The high measles seroprevalence estimate using residual specimens is similar to other, although limited, measles serosurveys among adults in India. On the other hand, our rubella seroprevalence estimates are, on average, higher than other rubella serosurveys from India [11]. Measles serosurveys among adults are not commonly conducted because susceptibility tends to be low among adults (although recent evidence points to more adult susceptibility as countries move toward measles elimination [7]), and there is no routine measles IgG testing at a scale similar to what is done for rubella IgG testing among women. Two adult measles serosurveys conducted in India among young adults aged 18 − 23 years at academic institutions in Pune and Mumbai found seroprevalence of 87% and 91%, respectively [12, 13]. Another measles serosurvey among individuals 1 − 60 years in Chandigarh estimated seropositivity of 93% or higher for individuals above 15 years of age [14]. In contrast, there are many published rubella seroprevalence studies in India, specifically among pregnant women. A review published in 2012 by Dewan et al. [11] estimated rubella seronegativity from 26 studies ranged from 10–47% for non-pregnant females (i.e., 90–53% seroprevalence) and 5–46% (i.e., 95–54% seroprevalence) for pregnant females. This review highlights the heterogeneity of endemic rubella virus transmission across India [15]. Estimates in India since 2012 have generally fallen in this range, with studies reporting 80–85% rubella seroprevalence [16–19]. Our estimates using community and residual specimens are higher than most prior serosurveys (>90% rubella seroprevalence) but still within the expected range. Spatial variability in both endemic rubella virus transmission and private-sector rubella vaccination for children as well as women of reproductive age are the likely drivers of these differences. One-quarter of female participants in our community serosurvey reported receiving RCV. We do not know how this compares to individuals attending the facilities or to other districts; however, in a study of female medical and nursing students in Rajasthan, no individuals reported rubella vaccination in adolescence [16].

Our second aim was to assess whether subgroups at the health facilities, such as male patients or ANC patients, were broadly representative of the population and if there were differences in seroprevalence across these populations. Of particular interest was to compare rubella seroprevalence estimates from women attending ANC clinics, an easy-to-access and generally healthy population, to all other male and female patients. No differences in measles or rubella seroprevalence were observed between female ANC attendees and male and non-ANC female patients. This suggests rubella seroprevalence findings from women attending ANC clinics are representative of all adults in the same age range, as assumed in previous rubella serosurveys nested in ANC samples. Measles seroprevalence did not differ by sex, although males had significantly lower rubella seroprevalence compared to females. As rubella virus circulated when these adults were children (~2008 and earlier given the average age of rubella infection is 5 years old and the youngest adults in the survey were 15 years old in 2018), both men and women should have been infected at similar rates. Differences in rubella seropositivity by sex have been observed in other studies of adolescents and young adults [13, 20]. Higher rubella seroprevalence among women may be related to private sector vaccine use among women of childbearing age and either providers preferentially offering the vaccine to women and/or families seeking vaccination for their daughters relative to sons.

This study had several limitations. Although the community serosurvey was designed to be representative of Palghar District, Maharashtra, the residual specimens were collected from only two sub-district hospitals, both located in rural settings. As such, the facility-based findings and its comparison with the community serosurvey, may not be representative of other parts of the district. The number of residual specimens collected from males and ANC attendees was small, so we did not have the power to detect the differences unless they exceeded 25% between groups. The patients whose residual specimens were collected were considerably younger than the community participants; however, this imbalance was accounted for in analyses. Although we estimated prior vaccine receipt among adult females in the community survey, this information was not available for patients at the health facilities and it is unknown how vaccination status compares between these populations.

Residual specimens provide a valuable alternative to those collected in community-based serosurveys, which are often costly and resource-intensive. Use of residual specimens has been limited due to concerns these may be biased and not representative of the underlying community. However, there has been increased interest in the use of residual health facility specimens in recent years to rapidly measure SARS-CoV-2 seroprevalence and monitor trends [21]. Despite the potential usefulness of residual health facility specimens for estimating seroprevalence, there is limited evidence comparing residual and community surveys conducted in similar settings and time periods [8, 9, 22]. The similarity in measles and rubella seroprevalence between the community-based and facility serosurveys in Palghar District highlights the potential value of these specimens and provides one example of how facility specimens can be used to approximate community seroprevalence. Future research is needed to evaluate the utility of residual specimens in other settings and for other diseases.

## Supporting information

Prosperi et al. supplementary material Prosperi et al. supplementary material

## Data Availability

A subset of the key anonymized individual participant data collected during the study, along with a data dictionary, is available upon request made to the corresponding author, after approval of a proposal by the study core investigators with a signed data-access agreement.

## Supplementary material

For supplementary material accompanying this paper visit http://doi.org/10.1017/S0950268824001389. click here to view supplementary material

## References

[1] Winter AK, et al. (2018) Benefits and challenges in using seroprevalence data to inform models for measles and rubella elimination. Journal of Infectious Diseases 218(3), 355–364. 29562334 10.1093/infdis/jiy137 PMC6049004

[2] Hasan AZ, et al. (2021) Implementing Serosurveys in India: Experiences, lessons learned, and recommendations. American Journal of Tropical Medicine and Hygiene 105(6), 1608–1617. 34607310 10.4269/ajtmh.21-0401 PMC8641364

[3] Mutembo S, et al. (2018) Integrating blood collection within household surveys: Lessons learned from nesting a measles and rubella serological survey within a post-campaign coverage evaluation survey in Southern Province, Zambia. American Journal of Tropical Medicine and Hygiene 99(6), 1639–1642. 30277204 10.4269/ajtmh.18-0320 PMC6283518

[4] Shook-Sa BE, Boyce RM and Aiello AE (2020) Estimation without representation: Early severe acute respiratory syndrome coronavirus 2 seroprevalence studies and the path forward. Journal of Infectious Diseases 222(7), 1086–1089. 32750135 10.1093/infdis/jiaa429 PMC7454696

[5] Mirambo MM, et al. (2015) Serological makers of rubella infection in Africa in the pre vaccination era: A systematic review. BMC Research Notes 8, 716. 26602892 10.1186/s13104-015-1711-x PMC4659241

[6] Trentini F, et al. (2017) Measles immunity gaps and the progress towards elimination: A multi-country modelling analysis. Lancet Infectious Diseases 17(10), 1089–1097. 28807627 10.1016/S1473-3099(17)30421-8

[7] Graham M, et al. (2019) Measles and the canonical path to elimination. Science 364(6440), 584–587. 31073065 10.1126/science.aau6299 PMC7745123

[8] Kugeler KJ, et al. (2022) Assessment of SARS-CoV-2 seroprevalence by community survey and residual specimens, Denver, Colorado, July-august 2020. Public Health Reports 137(1), 128–136. 34752156 10.1177/00333549211055137 PMC8721766

[9] Bajema KL, et al. (2021) Comparison of estimated severe acute respiratory syndrome coronavirus 2 seroprevalence through commercial laboratory residual sera testing and a community survey. Clinical Infectious Diseases 73(9), e3120–e3123. 33300579 10.1093/cid/ciaa1804 PMC7799302

[10] Murhekar MV, et al. (2022) Evaluating the effect of measles and rubella mass vaccination campaigns on seroprevalence in India: A before-and-after cross-sectional household serosurvey in four districts, 2018–2020. Lancet Global Health 10(11), e1655–e1664. 36240831 10.1016/S2214-109X(22)00379-5 PMC9579355

[11] Dewan P and Gupta P (2012) Burden of congenital rubella syndrome (CRS) in India: A systematic review. Indian Pediatrics 49(5), 377–399. 22700664 10.1007/s13312-012-0087-4

[12] Karade S, et al. (2019) Measles, mumps, and rubella: A cross-sectional study of susceptibility to vaccine-preventable diseases among young people in India. Medical Journal, Armed Forces India 75(1), 70–73. 30705481 10.1016/j.mjafi.2018.12.010 PMC6349678

[13] Gohil DJ, et al. (2016) Seroprevalence of measles, mumps, and rubella antibodies in college students in Mumbai, India. Viral Immunology 29(3), 159–163. 26910764 10.1089/vim.2015.0070

[14] Mathew JL, et al. (2022) Measles seroprevalence in persons over one year of age in Chandigarh, India. Human Vaccines & Immunotherapeutics 18(6), 2136453. 36279515 10.1080/21645515.2022.2136453 PMC9746381

[15] Metcalf CJ, et al. (2011) The epidemiology of rubella in Mexico: Seasonality, stochasticity and regional variation. Epidemiology and Infection 139(7), 1029–1038. 20843389 10.1017/S0950268810002165 PMC3884048

[16] Shekhar S, et al. (2020) Rubella seroprevalence among Indian female medical and nursing students at a tertiary care teaching institute and its correlation with socioeconomic status. Indian Journal of Community Medicine 45(2), 246–247. 32905212 10.4103/ijcm.IJCM_319_19 PMC7467205

[17] Muliyil DE, et al. (2018) Sero-prevalence of rubella among pregnant women in India, 2017. Vaccine 36(52), 7909–7912. 30448333 10.1016/j.vaccine.2018.11.013

[18] Koshy AK, Varghese JG and Issac J (2018) Seroprevalance of rubella in an urban infertility clinic - observations and challenges ahead. Journal of Human Reproductive Sciences 11(4), 384–387. 30787525 10.4103/jhrs.JHRS_16_18 PMC6333038

[19] Shanmugasundaram D, et al. (2021) Burden of congenital rubella syndrome (CRS) in India based on data from cross-sectional serosurveys, 2017 and 2019-20. PLoS Neglected Tropical Diseases 15(7), e0009608. 34297716 10.1371/journal.pntd.0009608 PMC8376255

[20] Poethko-Muller C and Mankertz A (2012) Seroprevalence of measles-, mumps- and rubella-specific IgG antibodies in German children and adolescents and predictors for seronegativity. PLoS One 7(8), e42867. 22880124 10.1371/journal.pone.0042867 PMC3412821

[21] Bobrovitz N, et al. (2021) Global seroprevalence of SARS-CoV-2 antibodies: A systematic review and meta-analysis. PLoS One 16(6), e0252617. 34161316 10.1371/journal.pone.0252617 PMC8221784

[22] Kelly H, et al. (2002) A random cluster survey and a convenience sample give comparable estimates of immunity to vaccine preventable diseases in children of school age in Victoria, Australia. Vaccine 20(25–26), 3130–3136. 12163264 10.1016/s0264-410x(02)00255-4

