# Supplementary material for: Measles and rubella seroprevalence in adults using residual blood samples from health facilities and household serosurveys in Palghar District, Maharashtra, India, 2018 – 2019
Source: Epidemiol Infect. 2024 Dec 6;152:e161. doi: 10.1017/S0950268824001389 (PMC11626445; doi:10.1017/S0950268824001389)
Supplement: Prosperi et al. supplementary material [file S0950268824001389sup001.docx]

***Epidemiology and Infection***

**Measles and rubella seroprevalence in adults using residual blood samples from health facilities and household serosurveys in Palghar District, Maharashtra, India, 2018-19**

Authors: Christine Prosperi, Alvira Z Hasan, Amy K Winter, Itta Krishna Chaaithanya, Neha R Salvi, Sanjay L Chauhan, Ragini N Kulkarni, Abhishek Lachyan, Poonam Gawali, Mitali Kapoor, Vaishali Bhatt, Ojas Kaduskar, Gururaj Rao Deshpande, Ignacio Esteban, R Sabarinathan, Velusamy Saravana Kumar, Shaun A Truelove, Muthusamy Santhosh Kumar, Jeromie W Vivian Thangaraj, Lucky Sangal, Sanjay M Mehendale, Gajanan N Sapkal, Nivedita Gupta, Kyla Hayford, William J Moss, Manoj V Murhekar

**Supplementary Tables and Figures**

Supplementary Table S1. Age of adult patients with residual specimens

|  | Median age in years (IQR) |
| --- | --- |
| Overall (N=650) | 27 (22, 35) |
| Female (N=476) | 26 (22, 32) |
| Male (N=174) | 30 (23, 40) |
| ANC attendees (N=167) | 24 (22, 27) |
| Male and non-ANC female patients (N=483) | 29 (23, 37.5) |

IQR, interquartile range

Supplemental Table S2. Seroprevalence of IgG antibodies against measles and rubella among adults 15 to 50 years with residual specimens

| Antigen | Overall (N=650) | By sex | | | By patient type | | |
| --- | --- | --- | --- | --- | --- | --- | --- |
|  |  | Female (N=476) | Male (N=174) | aOR^a^ (95% CI) | ANC attendees (N=167) | Male and non-ANC female patients (N=483) | aOR^b^  (95% CI) |
|  | % (95% CI) | % (95% CI) | % (95% CI) |  | % (95% CI) | % (95% CI) |  |
| Measles | 98.5 (97.5, 99.4) | 98.3 (97.2, 99.5) | 98.9 (97.3, 100) | 1.31 (0.32, 8.77) | 97.0 (94.4, 99.6) | 99.0 (98.0, 99.9) | 0.37 (0.07, 1.55) |
| Rubella | 91.5 (89.4, 93.8) | 92.6 (90.0, 95.0) | 88.5 (83.8, 93.2) | **0.54 (0.30, 0.99)** | 91.6 (87.4, 95.8) | 91.5 (89.0, 94.0) | 0.95 (0.47, 1.97) |

1. Logistic regression adjusted for age in years. Bold indicates p-value < 0.05.
2. Logistic regression adjusted for age in years and sex.

Supplementary Figure S1. Age distribution of adults with specimens collected, by survey type


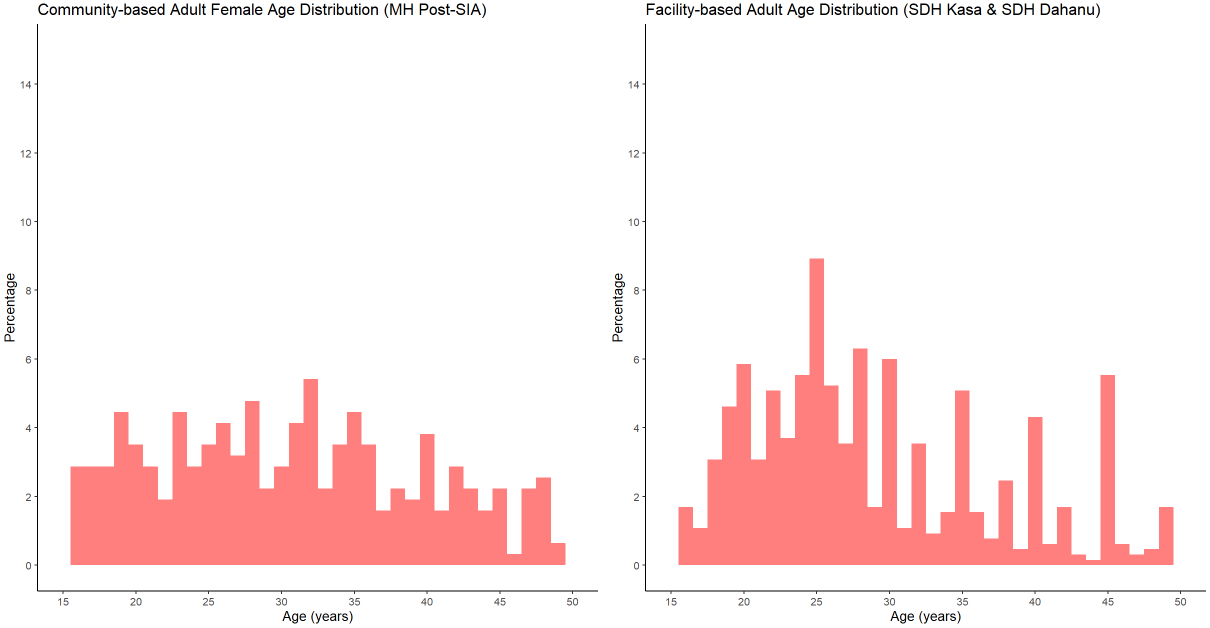


Median (IQR) age of adult females enrolled in community-based survey (N=314): 30.8 (23.3, 37.9).

Supplementary Table S3. Moran's I statistic results for measles and rubella seroprevalence estimated from post-SIA serosurvey conducted in Palghar District, Maharashtra

|  | **Measles** | **Rubella** |
| --- | --- | --- |
| Moran's Index, (SD) | 0.1 (1) | -0.09 (-0.48) |
| Expected Index | -0.03 | -0.03 |
| Variance | 0.017 | 0.013 |
| *p-value (Monte Carlo simulation, 1000 permutations)* | 0.139 | 0.718 |
